# Association between Source-Specific Particulate Matter Air Pollution and hs-CRP: Local Traffic and Industrial Emissions

**DOI:** 10.1289/ehp.1307081

**Published:** 2014-04-22

**Authors:** Frauke Hennig, Kateryna Fuks, Susanne Moebus, Gudrun Weinmayr, Michael Memmesheimer, Hermann Jakobs, Martina Bröcker-Preuss, Dagmar Führer-Sakel, Stefan Möhlenkamp, Raimund Erbel, Karl-Heinz Jöckel, Barbara Hoffmann, on behalf of the Heinz Nixdorf Recall Study Investigative Group.

**Affiliations:** 1 IUF-Leibniz Research Institute for Environmental Medicine, Düsseldorf, Germany; 2 Institute for Medical Informatics, Biometry and Epidemiology, University Hospital, University Duisburg-Essen, Essen, Germany; 3 Rhenish Institute for Environmental Research (RIU), Köln, Germany; 4 University Hospital of Essen, Essen, Germany; 5 Department of Cardiology, Hospital Bethanien Moers, Moers, Germany; 6 West German Heart Center, University Hospital of Essen, Essen, Germany; 7 Medical Faculty, Heinrich Heine University of Düsseldorf, Düsseldorf, Germany

## Abstract

Background: Long-term exposures to particulate matter air pollution (PM_2.5_ and PM_10_) and high traffic load have been associated with markers of systemic inflammation. Epidemiological investigations have focused primarily on total PM, which represents a mixture of pollutants originating from different sources.

Objective: We investigated associations between source-specific PM and high-sensitive C-reactive protein (hs-CRP), an independent predictor of cardiovascular disease.

Methods: We used data from the first (2000–2003) and second examination (2006–2008) of the Heinz Nixdorf Recall study, a prospective population-based German cohort of initially 4,814 participants (45–75 years of age). We estimated residential long-term exposure to local traffic- and industry-specific fine particulate matter (PM_2.5_) at participants’ residences using a chemistry transport model. We used a linear mixed model with a random participant intercept to estimate associations of source-specific PM and natural log-transformed hs-CRP, controlling for age, sex, education, body mass index, low- and high-density lipoprotein cholesterol, smoking variables, physical activity, season, humidity, and city (8,204 total observations).

Results: A 1-μg/m^3^ increase in total PM_2.5_ was associated with a 4.53% increase in hs-CRP concentration (95% CI: 2.76, 6.33%). hs-CRP was 17.89% (95% CI: 7.66, 29.09%) and 7.96% (95% CI: 3.45, 12.67%) higher in association with 1-μg/m^3^ increases in traffic- and industry-specific PM_2.5_, respectively. Results for PM_10_ were similar.

Conclusions: Long-term exposure to local traffic-specific PM (PM_2.5_, PM_10_) was more strongly associated with systemic inflammation than total PM. Associations of local industry-specific PM were slightly stronger but not significantly different from associations with total PM.

Citation: Hennig F, Fuks K, Moebus S, Weinmayr G, Memmesheimer M, Jakobs H, Bröcker-Preuss M, Führer-Sakel D, Möhlenkamp S, Erbel R, Jöckel KH, Hoffmann B, Heinz Nixdorf Recall Study Investigative Group. 2014. Association between source-specific particulate matter air pollution and hs-CRP: local traffic and industrial emissions. Environ Health Perspect 122:703–710; http://dx.doi.org/10.1289/ehp.1307081

## Introduction

Long-term exposure to fine particulate matter (PM_2.5_; ≤ 2.5 μm in aerodynamic diameter) air pollution and long-term exposure to high traffic load at the residence have been associated with increased cardiovascular morbidity and mortality (Brook et al. 2010; Brunekreef et al. 2009). Furthermore, it has been hypothesized that long-term exposure to PM_2.5_ might lead to the development and progression of atherosclerosis (Bauer at al. 2010; Künzli et al. 2010; Sun et al. 2005), which has a strong inflammatory component (Libby 2012). High-sensitive C-reactive protein (hs-CRP) is a widely used marker for systemic inflammation and an independent predictor of cardiovascular disease (Ridker et al. 2002). Although short-term exposure studies have reported an association between PM_2.5_ and hs-CRP (Delfino et al. 2008; Seaton et al. 1999), evidence from epidemiological studies of the long-term effects of air pollution on inflammatory markers has been inconsistent (Diez Roux et al. 2006; Forbes et al. 2009; Hoffmann et al. 2009; Panasevich et al. 2009). One possible reason for the observed inconsistencies between studies relates to the relative toxicity of the different components or sources of the total PM mixture (Kelly and Fussell 2012), which may be greater for traffic-related emissions and metal-rich PM than other PM mixtures (Sarnat et al. 2008; Stanek et al. 2011). Several studies have reported stronger associations of cardiovascular outcomes with traffic-related PM_2.5_ than with total PM_2.5_ (Health Effects Institute 2010). However, information regarding associations between source-specific PM_2.5_ and markers of inflammation is limited (Rückerl et al. 2011).

We aimed to estimate associations between source-specific PM and hs-CRP to gain more insight into whether the toxicity of PM air pollution varies depending on its source. To that end, we applied a chemistry transport model, using input data from detailed emission inventories, meteorology, and land use variables, to estimate the surface concentration of air pollutants. We estimated source-specific PM concentrations by estimating total PM concentrations under alternative emissions scenarios in which contributions from individual source categories were set to zero. Because our study area was located in the Ruhr Area, an urban and industrial area in North Rhine-Westphalia, Germany, we focused on two anthropogenic sources, namely local traffic and local industry.

## Methods

*Study design*. We used data from the baseline and first follow-up examination of the Heinz Nixdorf Recall (HNR) study, a population-based prospective cohort study. The overall study group was randomly selected from registries of the local residents of three large adjacent cities (Mülheim, Essen, and Bochum) in the Ruhr Area (also referred to as the HNR study area) and was 45–75 years of age at baseline. A total of 4,814 participants completed the baseline visit in 2000–2003, and 4,157 completed the first follow-up study visit during 2006–2008. The study design has been described in detail elsewhere (Schmermund et al. 2002). The study was approved by the institutional ethics committees of the University Duisburg-Essen and the University Hospital of Essen and followed strict internal and external quality assurance protocols. Assessment for both baseline and first follow-up examination included a self-administered questionnaire, face-to-face interviews for personal risk factor assessment, clinical examinations, and comprehensive laboratory tests according to standard protocols. All participants gave informed consent.

*Environmental exposures*. Air pollution. We used the validated time-dependent three-dimensional chemistry transport model European Air Pollution Dispersion chemical transport model (EURAD-CTM) (Ebel et al. 1997; Hass et al. 1993; Memmesheimer et al. 2004; Schell et al. 2001) to predict daily mass concentrations of PM_10_ (≤ 10 μm in aerodynamic diameter) and PM_2.5_ on a horizontal grid resolution of 1 km. The EURAD-CTM model system is a multilayer, multigrid model system for the simulation of transport, chemical transformation, and deposition of tropospheric constituents (Büns et al. 2012) (for details see Supplemental Material, “Air Pollution Exposure Assessment,” pp. 2–4). The multigrid system defines a sequential nesting of four horizontal grid sizes from Europe (grid size of 125 km) over central Europe (25 km), North Rhine-Westphalia in Germany (5 km) to the southwestern part of the Ruhr Area (Duisburg-Mülheim-Essen-Bochum) (1 km) (Büns et al. 2012, Memmesheimer et al. 2004). The emission input of the model is structured with respect to different source categories according to the Selected Nomenclature for Sources of Air Pollution (SNAP-97; European Environment Agency 2002), which, for example, includes traffic and industrial sources as different source categories. Output of the EURAD-CTM calculations includes a set of chemical compounds such as atmospheric particle mass, number density, and particle size distribution and concentration of atmospheric gases, photooxidants, and a set of volatile organic compounds on a daily basis for each grid.

For sensitivity studies or emission scenarios, the emission input into the EURAD-CTM can be modified or set to zero for each source category separately to study the impact of certain source categories on the concentration values (Hebbinghaus et al. 2009). We applied this method to investigate the impact of local traffic- and local industrial sources within the Ruhr Area (1 km). To do so, we first performed three different EURAD-CTM runs, which were independent of each other and differed only by emission input: *a*) complete emission input, including all sources, which we refer to as total PM (PM_ALL_); *b*) emission input, excluding emissions from local road traffic within the Ruhr Area (i.e., corresponding emission factors were set to zero), which we refer to as PM_noTRA_; and *c*) emission input, excluding emissions from local industry within the Ruhr Area, which we refer to as PM_noIND_. We then defined the concentration of local traffic-specific PM as PM_TRA_ = PM_ALL_ – PM_noTRA_ and the concentration of local industry-specific PM as PM_IND_ = PM_ALL_ – PM_noIND_. Because all scenarios (PM_ALL_, PM_noTRA_, PM_noIND_) were based on the same mass model equation and differed only by emission input, PM_TRA_ is an estimate of PM concentrations due solely to local traffic, and PM_IND_ is an estimate of PM concentrations due solely to local industrial sources.

The HNR study area covers a region of approximately 600 km^2^ within the Ruhr Area. Hence, we were able to assign daily PM concentrations for each 1-km^2^ grid cell to the participants’ addresses (ArcView, version 9.2; ESRI, Redlands, CA, USA). We then calculated residential long-term exposure as a 365-day average, referring to 365 days before blood draw (for baseline and first follow-up examination). Short- and medium-term residential exposure PM refers to the average PM concentration of the last 7 and the last 28 or 91 days before blood draw, respectively.

Meteorological data. Short-term temperature, wind speed (in meters per second) (north-to-south wind and east-to-west wind), and humidity refer to a moving average within 7 days before blood draw.

Noise. Long-term road noise was modeled according to the European Union directive (2002/49/EC) (European Commission 2002) for the year 2005 as the weighted 24-hr mean (L_den_) and weighted nighttime (2200–0600 hours) mean (L_night_), using the maximum noise value in a 10-m buffer around each participant’s address. For 60 participants, noise values were imputed from isophone bands. Noise values were investigated as categories of 5 dB(A), with the exception of the lowest category (0–45 dB(A).

Traffic. We assessed distance (in meters) to high-traffic roads [i.e., roads with a traffic count of > 26.000 vehicles/day (upper quintile of traffic density)], using official digitized maps with a precision of at least 0.5 m. The reference line was the median strip between the oncoming traffic lanes.

*Measurement of hs-CRP*. As a marker of inflammation, we measured serum hs-CRP using an automated nephelometer (BN-II; Dade-Behring Inc., Deerfield, IL, USA). All analyses were performed in the central laboratory of the University Hospital of Essen, following a standard procedure.

*Definition of covariates*. Individual socioeconomic status (SES) was defined by years of education. We classified education according to the International Standard Classification of Education as total years of formal education (United Nations Educational, Scientific, and Cultural Organization 1997), using three categories [≤ 10, 11–13, and ≥ 14 (reference) years of education] for the analysis. To assess neighborhood-based SES, the cities were divided into 106 neighborhoods according to administrative boundaries, with a median size of 11,263 inhabitants [interquartile range (IQR), 7,875–16,022]. Neighborhood-based SES was provided by the local census authorities and included unemployment rate, welfare, mean income, retired population rate, population density, and residential stability (Dragano et al. 2009). Smoking status was defined as current, former, and never-smoker, based on the past year. Cumulative smoking exposure was assessed for former and current smokers using pack-years, accounting for time periods of nonsmoking. Environmental tobacco smoke (ETS) was defined as any passive tobacco smoke exposure at home and/or at work (yes/no). Physical activity was assessed as times per week and categorized in three groups (< 1, 1–3, and > 3 times/week). Alcohol consumption was operationalized as drinks per week. Anthropometric measurements (height, weight) were conducted according to standardized protocols. Body mass index (BMI) was calculated as kilograms per meter squared. Diabetes mellitus (DM) was defined as prior physician diagnosis of diabetes or taking an antidiabetic drug or having a blood glucose ≥ 200 mg/dL or having a fasting blood glucose ≥ 126 mg/dL. Standard enzymatic methods were used to measure total cholesterol, high-density lipoprotein cholesterol (HDL-C). Low-density lipoprotein cholesterol (LDL-C) was measured directly (Schmermund et al., 2002). Current medications (i.e., statins) were coded according to the Anatomical Therapeutic Chemical Classification Index of the World Health Organization (WHO Collaborating Centre for Drug Statistics Methology 2014). All characteristics were updated at the first follow-up study visit. Coronary heart disease (CHD) at baseline was defined as a self-reported history of a myocardial infarction or coronary intervention. Incident CHD during follow-up was based on self-reported incident coronary events that met predefined study criteria (Schmermund et al. 2002), confirmed with medical records by a study end point committee (Erbel et al. 2010). We used indicator variables to model season (spring, summer, fall, or winter according to meteorological seasons), city (Mülheim, Essen, or Bochum), and a created area variable (north, center, or south) based on ZIP codes (Hoffmann et al. 2006), which was equivalent to low, medium, and high neighborhood-based SES.

*Analytical strategy*. There were 8,634 observations from 4,793 participants with complete information on exposure and hs-CRP. We excluded participants with acute infections or acute exacerbations of inflammatory disease—defined by hs-CRP > 10 mg/dL—from the study population (*n* = 5). The final data set with complete information on covariates used for analysis included 8,204 observations (from 4,665 participants, of which 3,539 supplied repeated measurements). We performed repeated measurement analysis to investigate the association between total and source-specific PM and hs-CRP using linear mixed models including a random participant intercept to account for the correlation of repeated measures. We assumed a compound symmetry covariance structure, that is, equal variation of hs-CRP at both measurements (Box et al. 1994). hs-CRP was log-transformed (natural logarithm), and thus results are presented as the percent-change of hs-CRP {100 × [exp(β) – 1]}.

Model 1 included a minimal adjustment of age, sex, education, BMI, LDL-C, and HDL-C. In model 2, we additionally included lifestyle variables (smoking status, pack-years, ETS, physical activity, alcohol consumption) that predicted hs-CRP with *p* < 0.10. In model 3, we additionally included meteorological variables (season, temperature, humidity, wind speed) that predicted hs-CRP with *p* < 0.10. Our main analysis model (Main) therefore included age, sex, education, BMI, LDL-C, HDL-C, smoking status, pack-years of smoking, ETS, physical activity, indicator variables for summer and fall (winter and spring served as reference), humidity, plus city, which was included to capture spatial (unmeasured) confounding. We confirmed that covariate–outcome relationships for continuous variables (age, BMI, LDL-C, HDL-C, pack-years of smoking, humidity) did not significantly depart from linearity based on likelihood ratio tests comparing models with and without squared terms (*p* > 0.05).

*Effect modification*. We evaluated effect modification by modeling interaction terms between each exposure (modeled as a continuous variable) and age (≤ 65 years, > 65 years), sex (males, females), ETS, CHD, intake of statins (yes, no), area (north, center, or south), city of residence (Mülheim, Essen, or Bochum), and wind direction (east vs. west, or north vs. south). Each potential modifier was defined according to its value at the study visit when the exposure and hs-CRP were measured. In addition, we investigated the potential modifying role of PM_IND_ (dichotomized at the third quartile) on PM_TRA_ and vice versa.

*Sensitivity analysis*. To evaluate the robustness of our main analysis model, we performed a series of models that included additional covariates. To take overall exposure levels of PM exposure into account when analyzing source-specific associations, we adjusted the source-specific models of PM_TRA_ and PM_IND_ for PM_noTRA_ and PM_noIND_ (i.e., PM from all other sources). We added indicators of neighborhood-based SES (e.g., unemployment rate) because previous studies reported an independent effect on various cardiovascular disease–related outcomes (Foraker et al. 2010). Furthermore we added covariates known to be associated with cardiovascular disease or with systemic inflammation—such as hypertension, diabetes, and intake of statins—to investigate the robustness of our main analysis model. To account for small-scale variation in traffic-related exposures, we additionally adjusted for traffic indicator variables and road traffic noise. Furthermore, we investigated short- and medium-term (7-day and 28- or 91-day average) exposure to PM.

To evaluate the clinical relevance of exposure effects, we dichotomized hs-CRP as ≤ 0.3 or > 0.3 mg/dL, a cut point commonly used to denote an increased cardiovascular risk, and performed multivariable logistic regressions.

## Results

*Study population*. The study population available for main analysis (*n* = 8,204 observations) (Table 1) included 4,379 participants (49.3% males; 59.7 ± 7.8 years of age) at the baseline examination and 3,825 participants (49.5% males) at the first follow-up examination. Excluded observations (*n* = 430) were due to missing data on the outcome, exposure, or main analysis covariates and did not show systematic differences regarding exposure, outcome, or covariates (data not shown). Mean values for BMI, systolic blood pressure, and HDL-C did not change remarkably over time (Table 1), whereas LDL-C changed from borderline-high values at baseline to relatively normal values at the first follow-up. However, over time we observed fewer current smokers, and the prevalence of diabetes mellitus and statin intake increased.

**Table 1:** Characteristics of the HNR study population at the time of the baseline (2000–2003) and first follow-up (2006–2008) study examinations.

Characteristic^*a*^	Baseline (2000–2003) (*n* = 4,379)	First follow-up (2006–2008) (*n* = 3,825)
Age (years)	59.7 ± 7.8	64.5 ± 7.6
Sex (male)	2,157 (49.3)	1,895 (49.5)
hs-CRP [mg/dL]^*b*^	0.15 (0.26)	0.15 (0.22)
BMI (kg/m^2^)	27.9 ± 4.6	28.3 ± 4.8
LDL-C (mg/dL)	145.7 ± 36.3	130.9 ± 34.7
HDL-C (mg/dL)	57.9 ± 16.9	59.8 ± 16.2
Systolic blood-pressure (mmHg)	132.7 ± 20.6	134.2 ± 19.8
CHD	300 (6.9)	337 (8.8)
Diabetes mellitus	611 (14.0)	729 (19.1)
Smoking status
Current	1,022 (23.9)	678 (17.7)
Former	1,515 (34.6)	1,533 (40.1)
Never	1,842 (42.1)	1,614 (42.2)
Pack-years of smoking	16.6 ± 25.4	16 ± 24.4
ETS	1,573 (35.9)	1,352 (35.3)
Intake of statins	468 (10.7)	764 (20.0)
Education
Low	493 (11.3)	386 (10.1)
Medium	2,438 (55.7)	2,145 (56.1)
High	1,448 (33.1)	1,294 (33.8)
Physical activity
Low	2,261 (51.6)	1,859 (48.6)
Medium	478 (10.9)	448 (11.7)
High	1,640 (37.5)	1,518 (39.7)
Unemployment rate in neighborhood	12.5 ± 3.4	12.5 ± 3.4
Humidity (%)	6.6 ± 2.4	6.5 ± 2.0
Ozone (μg/m^3^)	36.6 ± 19.2	36.9 ± 16.0
West wind (dominant)	1,291 (29.5)	956 (25.0)
North wind (dominant)	3,461 (79.0)	2,956 (77.3)
Season
Spring	1,189 (27.7)	1,054 (27.6)
Summer	1,240 (28.3)	875 (22.9)
Fall	1,032 (23.6)	939 (24.5)
Winter	918 (21.0)	957 (25.0)
City
Mülheim	1,626 (37.1)	1,420 (37.1)
Essen	1,466 (33.5)	1,292 (33.8)
Bochum	1,287 (29.4)	1,113 (29.1)
Proximity to traffic (m)	1019.2 ± 807.9	1022.8 ± 809.8
^***a***^Values are mean ± SD or *n* (%) unless otherwise indicated. ^***b***^Values are median (interquartile range).		

*Exposure*. The residential 365-day mean concentration of PM_2.5ALL_ was 16.72 ± 1.60 μg/m^3^ at baseline examination (Table 2). The amount from PM_2.5TRA_ was 4.8% with a mean concentration of 0.81 ± 0.24 μg/m^3^, whereas PM_2.5IND_ contributed 10.2% with a mean concentration of 1.70 ± 0.94 μg/m^3^. In contrast to PM_2.5_, mean concentrations for PM_10_ were noticeably higher for PM_10ALL_ (19.68 ± 2.12 μg/m^3^) and PM_10IND_ (2.47 ± 1.46 μg/m^3^), although mean concentrations of PM_10TRA_ were similar to PM_2.5TRA_ (0.81 ± 0.24 μg/m^3^). At the first follow-up examination, PM concentrations were lower but showed similar patterns.

**Table 2:** Distributions of residential concentrations of 365-day exposure to particulate matter (PM_2.5ALL_, PM_10ALL_, PM_2.5TRA_, PM_10TRA_, PM_2.5IND_, and PM_10IND_) for baseline (2000–2003) and first follow-up (2006–2008) examination.

Exposure [μg/m^3^]; examination	Mean ± SD	Minimum	Maximum	IQR	Percentage of PM_ALL_
PM_2.5ALL_
1	16.72 ± 1.60	13.28	22.38	2.39	100.0
2	15.63 ± 1.35	12.72	21.16	2.04	100.0
PM_10ALL_
1	19.68 ± 2.12	15.60	28.15	3.12	100.0
2	18.08 ± 1.76	14.74	25.76	2.74	100.0
PM_2.5TRA_
1	0.81 ± 0.24	0.23	1.71	0.31	4.8
2	0.57 ± 0.16	0.17	1.26	0.22	3.7
PM_10TRA_
1	0.81 ± 0.24	0.22	1.76	0.32	4.1
2	0.56 ± 0.17	0.15	1.24	0.22	3.1
PM_2.5IND_
1	1.70 ± 0.94	0.36	5.96	1.37	10.2
2	1.51 ± 0.83	0.37	5.58	1.31	9.7
PM_10IND_
1	2.47 ± 1.46	0.51	9.32	2.09	12.6
2	2.09 ± 1.21	0.47	8.18	1.90	11.6

**Figure 1:**
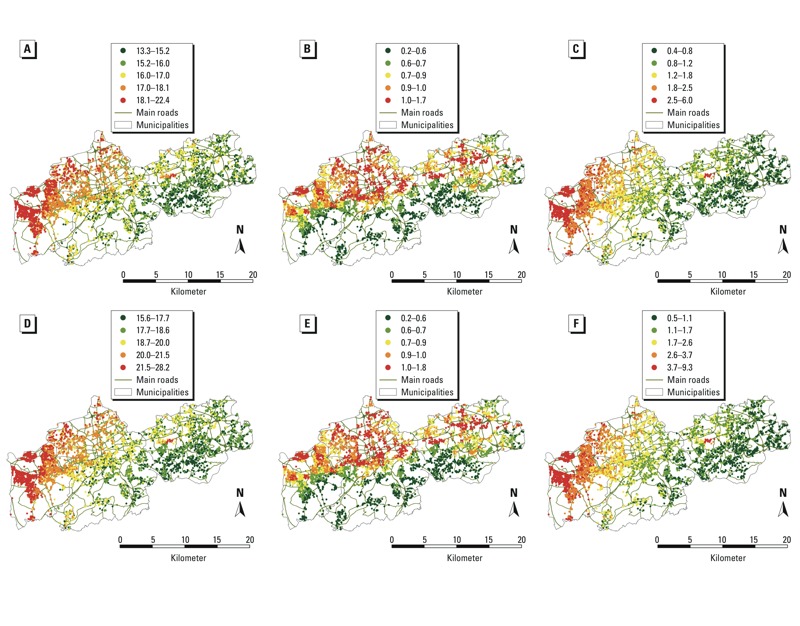
Distribution of individualized residential 365-day exposure to PM within the HNR study area, presented in five categories based on quintiles of distribution. (*A*) PM_2.5ALL_,, (*B*) PM_2.5TRA_, (*C*) PM_2.5IND_, (*D*) PM_10ALL_, (*E*) PM_10TRA_, and (*F*) PM_10IND_ for the study population at baseline examination (2000–2003).

Spatial distributions of residential 365-day mean concentrations of exposure show a decreasing west-to-east-gradient in the HNR study area for PM_ALL_ (PM_2.5_, PM_10_) and PM_IND_ (Figure 1A,C,D,F), whereas PM_TRA_ was distributed more homogeneously among cities, but clearly showed a decreasing north-to-south gradient (Figure 1B,E). The similarities in spatial gradients were reflected in the correlation structure: PM_2.5ALL_ and PM_2.5IND_ were strongly correlated, and PM_2.5ALL_ and PM_2.5TRA_ were only moderately correlated (Pearson correlation coefficient ρ = 0.89 and ρ = 0.40, respectively) (Table 3); PM_2.5IND_ and PM_2.5noIND_, and PM_2.5TRA_ and PM_2.5noTRA_ were moderately correlated (ρ = 0.54 and ρ = 0.27, respectively). PM_2.5ALL_ and PM_10ALL_ were strongly correlated (ρ = 0.99); therefore, correlation patterns for PM_10_ were very similar to those for PM_2.5_ (data not shown).

**Table 3:** Correlation coefficient between individualized residential concentrations of 365-day exposure to PM (PM_ALL_, PM_TRA_, PM_IND_, PM_noTRA_, PM_noIND_) for the baseline examination.

Exposure	PM_2.5TRA_	PM_2.5IND_	PM_2.5noTRA_	PM_2.5noIND_
PM_2.5ALL_	0.40	0.89	0.99	0.87
PM_2.5TRA_	1.00	0.24	0.27	0.48
PM_2.5IND_		1.00	0.90	0.54

*Association of source-specific PM with hs-CRP.* In our main analysis model, we estimated positive associations between hs-CRP and 1-μg/m^3^ increases in PM_2.5ALL_ (4.53% higher hs-CRP; 95% CI: 2.76, 6.33%) and PM_2.5TRA_ (17.89% higher hs-CRP; 95% CI: 7.66, 29.09%) (Table 4). The association between hs-CRP and PM_2.5IND_ (7.96% higher; 95% CI: 3.45, 12.67%) was slightly stronger than for PM_2.5ALL._ In the models without adjustment for city (models 1–3), estimates for PM_ALL_ and PM_IND_ were lower. Overall, associations of hs-CRP with PM_10_ and PM_2.5_ showed similar patterns.

**Table 4:** Estimated percentage difference (95% CI) in hs-CRP per 1-μg/m^3^ increase in (source-specific) PM (*n* = 8,204 from 4,665 participants).

Exposure and model	ALL	TRA	IND
PM_2.5_
Model 1^*a*^	2.60 (1.19, 4.04)	19.46 (9.07, 30.83)	2.01 (–0.77, 4.87)
Model 2^*b*^	2.68 (1.26, 4.11)	17.59 (7.41, 28.73)	2.08 (–0.66, 4.90)
Model 3^*c*^	2.81 (1.39, 4.25)	18.70 (8.42, 29.95)	2.17 (–0.57, 5.00)
Main^*d*^	4.53 (2.76, 6.33)	17.89 (7.66, 29.09)	7.96 (3.45, 12.67)
Main per IQR^*e*^	11.17 (6.73, 15.79)	5.24 (2.32, 8.24)	11.06 (4.75, 17.76)
Main + nSES	4.47 (2.7, 6.26)	17.76 (6.92, 29.71)	–2.1 (–5.31, 1.22)
Main + DM, SysBP, statins (*n* = 8,197)	4.38 (2.62, 6.18)	16.85 (6.73, 27.92)	7.91 (3.39, 12.62)
PM_10_
Model 1^*a*^	1.63 (0.56, 2.71)	19.59 (9.39, 30.75)	1.1 (–0.71, 2.94)
Model 2^*b*^	1.73 (0.66, 2.82)	17.75 (7.75, 28.68)	1.19 (–0.60, 3.01)
Model 3^*c*^	1.84 (0.76, 2.92)	18.83 (8.73, 29.86)	1.25 (–0.54, 3.07)
Main^*d*^	3.30 (1.94, 4.69)	18.07 (8.02, 29.06)	4.60 (1.75, 7.53)
Main per IQR^*f*^	10.67 (6.16, 15.37)	5.46 (2.50, 8.51)	9.86 (3.70, 16.39)
Main + nSES	3.24 (1.85, 4.65)	18.01 (7.35, 29.73)	4.46 (1.59, 7.40)
Main + DM, SysBP, statins (*n* = 8,197)	3.19 (1.82, 4.57)	17.08 (7.13, 27.95)	4.55 (1.70, 7.48)
Abbreviations: DM, diabetes mellitus; nSES, neighborhood-based socioeconomic status; statins, intake of statins; SysBP, systolic blood pressure.^***a***^Model 1: age, sex, education, BMI, LDL-C, HDL-C. ^***b***^Model 2: model 1 plus smoking status, pack-years of smoking, ETS, physical activity. ^***c***^Model 3: model 2 plus season, humidity. ^***d***^Main: model 3 plus city of residence. ^***e***^PM_2.5ALL_, 2.39; PM_2.5TRA_, 0.31; PM_2.5IND_, 1.37 μg/m^3^. ^***f***^PM_10ALL_, 3.12; PM_10TRA_, 0.32; PM_10IND_, 2.09 μg/m^3^.			

On a population-based exposure distribution scale (using the IQR at baseline), hs-CRP was 11.06% higher (95% CI: 4.75, 17.76%) in association with an IQR increase in PM_2.5IND_ (1.37 μg/m^3^), and 11.17% higher (95% CI: 6.73, 15.79%) per IQR increase in PM_2.5ALL_ (2.39 μg/m^3^). A 1-IQR increase in PM_2.5TRA_ (0.31 μg/m^3^) was associated with 5.24% higher hs-CRP (95% CI: 2.32, 8.24%).

**Figure 2:**
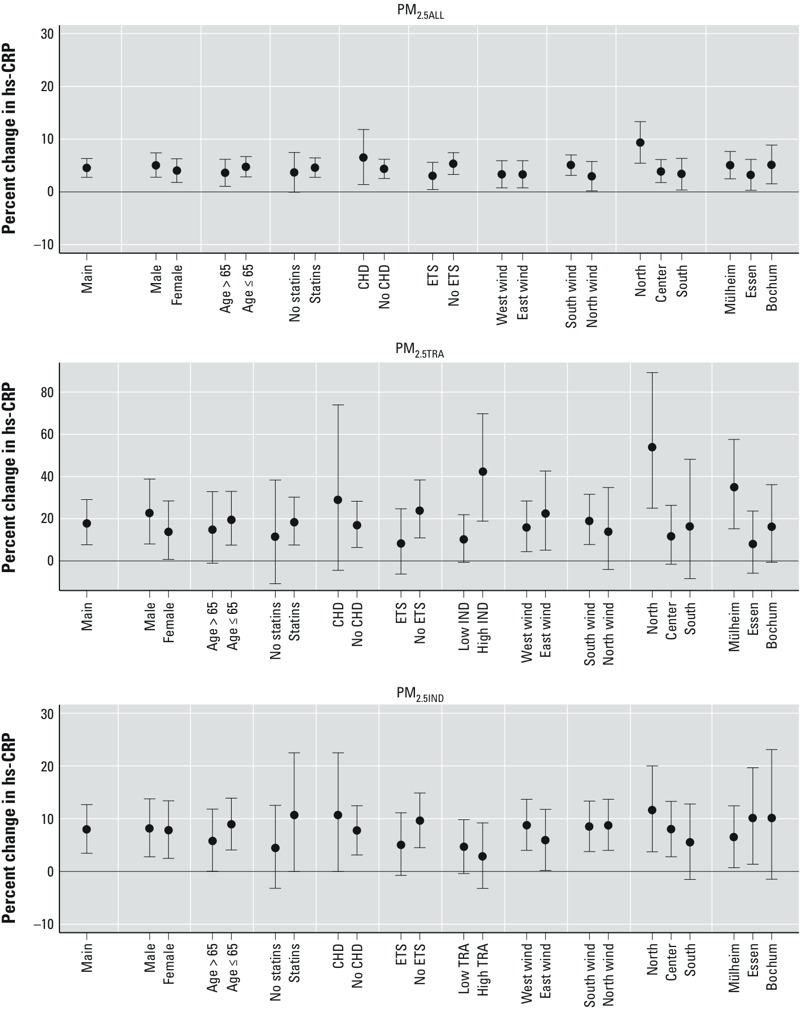
Effect modification for the association of PM_2.5ALL_, PM_2.5TRA_, and PM_2.5IND_ with hs-CRP presented as percent change (95% CI) per 1 μg/m^3^. Models adjusted for age, sex, education, BMI, LDL-C, HDL-C, smoking status, pack-years of smoking, ETS, physical activity, season, humidity, and city.

*Effect modification*. Generally, we observed slightly stronger associations among participants living in the north of the HNR study area compared with other areas, among participants not exposed (versus exposed) to ETS, among statin users, and among those with (vs. without) prevalent CHD at the corresponding examination, although CIs overlapped in most analyses and interaction *p*-values were between 0.1 and 0.5 (Figure 2). In males compared with females, we observed slightly higher effect PM_2.5ALL_ and PM_2.5TRA_ (*p*-values > 0.3). For PM_2.5TRA_, we also observed a stronger effect in participants exposed to higher levels of PM_2.5IND_ (*p* = 0.011) and in participants living in Mülheim (*p* = 0.051) and in Essen (*p* = 0.089). For PM_2.5IND_, we additionally observed slightly stronger associations for participants living in Essen (*p* = 0.108) and lower levels of traffic-specific PM exposure (*p* = 0.052). We did not find indications of effect modification by age or wind direction. Patterns of effect modification for PM_10_ were similar (data not shown).

*Sensitivity analysis*. Adjusting for the PM concentration of all other sources (PM_2.5noTRA_ or PM_2.5noIND_) attenuated associations of hs-CRP with 1-μg/m^3^ increases in PM_2.5TRA_ (5.50% higher; 95% CI: –5.38, 17.62%) and PM_2.5IND_ (3.44% higher; 95% CI: –1.45, 8.57%). In addition, the estimates became less precise. Corresponding estimates from two-pollutant models that were not adjusted for city were 12.87% (95% CI: 2.02, 24.89%) and –1.62% (95% CI: –4.67, 1.54%) for PM_2.5TRA_ and PM_2.5IND_, respectively. Adjusting for neighborhood-based SES indicators did not clearly change effect estimates for PM_2.5TRA_, but resulted in negative associations with PM_2.5IND_ (model fit did not improve) (Table 4; only adjustment for neighborhood unemployment rate is presented). Associations with PM_10_ were not affected by adjustment for neighborhood-based SES. Effect estimates among different sources and fractions of PM were robust toward an additional adjustment of health indicators, such as hypertension, diabetes, or intake of statins (Table 4). Additional adjustment of the main models for proximity to traffic or road traffic noise did not influence associations (data not shown).

**Figure 3:**
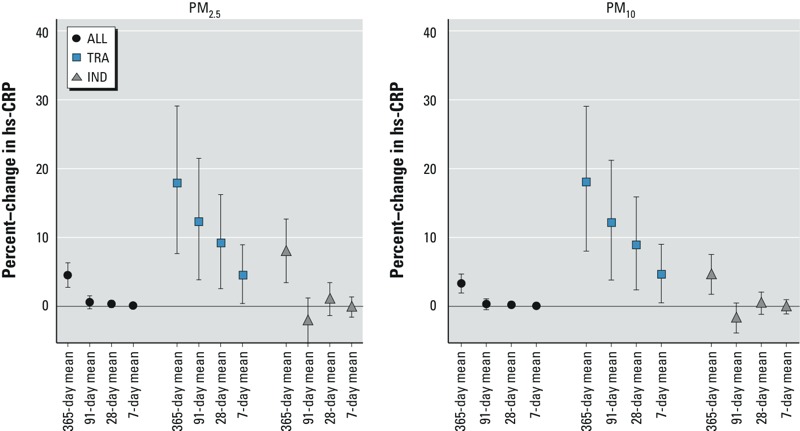
Effect estimates for mean short-term, mid-term, and long-term exposure to PM_2.5ALL_, PM_2.5TRA_, PM_2.5IND_, PM_10ALL_, PM_10TRA_, and PM_10IND_ on hs-CRP presented as percent change (95% CI) per 1 μg/m^3^. Models adjusted for age, sex, education, BMI, LDL-C, HDL-C, smoking status, pack-years of smoking, ETS, physical activity, season, humidity, and city.

Associations between hs-CRP and long-term exposures (averaged over 1 year) remained robust after adjustment for short-term exposure (averaged over 7 days) and increased slightly after adjustment for medium-term exposures (averaged over 91 or 28 days) (data not shown). There was also no indication of independent associations of hs-CRP with short- or medium-term exposures to PM_ALL_ or PM_IND_ (Figure 3), whereas for PM_TRA_ the positive association with hs-CRP increased with longer time windows of PM_TRA_ exposure.

In terms of clinical relevance, the odds of hs-CRP > 0.3 mg/dL (the highest cardiovascular risk group) was increased in association with 1-μg/m^3^ increases in PM_2.5ALL_ [odds ratio (OR) = 1.09; 95% CI: 1.02, 1.16], PM_2.5TRA_ (OR = 1.43; 95% CI: 1.02, 2.00), and PM_2.5IND_ (OR = 1.12; 95% CI: 0.96, 1.31).

## Discussion

To our knowledge, this is the first study to analyze the association of long-term exposure to source-specific PM with a marker of subclinical systemic inflammation (hs-CRP) in the general population. Applying a novel method to estimate long-term exposure to source-specific PM (PM_2.5_, PM_10_), we observed that PM from local road traffic was more strongly associated with hs-CRP than total PM, independent of short-term exposures. The association with industry-specific PM was slightly stronger, but not significantly different from the association with total PM. We observed predominantly similar patterns in concentration and effect estimates for PM_2.5_ and PM_10_. These findings were most likely due to the almost perfect correlation of 0.99, because PM_2.5_ was included in PM_10_.

We were able to confirm previously reported long-term associations of total urban background PM with hs-CRP (Hoffmann et al. 2009) in this extended database of 8,204 observations from 4,665 participants. Our results strengthen those of previous studies reporting weak associations of medium- or long-term exposures to PM with CRP (Diez Roux et al. 2006; Zeka et al. 2006).

Little information, however, is currently available about the comparative toxicity of source-specific fractions of the PM mixture on physiological or clinical outcomes. Among others, positive associations have been reported for traffic-related PM and NO_2_ with diabetes (Krämer et al. 2010), coronary heart disease hospitalization and morbidity (Gan et al. 2011), mortality (Beelen et al. 2008), and inflammatory markers (Panasevich et al. 2009). Furthermore, several studies using traffic indicators, such as traffic-density or distance to a major road, have reported associations with coronary heart disease (Hoffmann et al. 2006), atherosclerosis (Hoffmann et al. 2007; Künzli et al. 2010), diabetes (Krämer et al. 2010), and myocardial infarction (Tonne et al. 2006). Bind et al. (2012) recently reported that short- and medium-term exposure to particle number, a measure of fresh traffic emissions, was positively associated with CRP and other markers of cardiovascular risk in a study of 704 highly selected elderly men.

These studies, however, lack the possibility of directly comparing the toxicity of different sources using comparable units.

We used a novel approach to estimate source-specific PM exposures, namely exposures to local traffic- and local industry-specific PM, which enabled us to directly compare associations between hs-CRP and PM attributable to different sources. On the one hand, we observed a west-to-east-gradient for PM_ALL_ and PM_IND_, consistent with the location of heavy industry in the west of the Ruhr Area. On the other hand, we observed a north-to-south-gradient for PM_TRA_ that was consistent with population density and the location of major arterial roads in the HNR study area. This finding was interesting because it indicated the different long-term spatial patterns of these two major PM sources within our study area as well as their potential different associations with hs-CRP. The estimated effect of a 1-μg/m^3^ increase in local traffic-specific PM was 4–6 times stronger than the estimated effect of a comparable increase in total PM. Although effect estimates of PM_IND_ were generally slightly higher than those of PM_ALL_, we were not able to detect a significant difference in the associations. Because of the high correlation between PM_IND_ and PM_ALL_ or PM_noIND_ and PM_ALL_, we were not able to clearly differentiate between industry-specific and total PM.

Associations with a population-based unit of exposure (i.e., IQR) were weaker for PM_TRA_ (IQR = 0.31 μg/m^3^) than for PM_ALL_ (IQR = 2.30 μg/m^3^), and comparable for PM_ALL_ and PM_IND_ (IQR = 1.37 μg/m^3^). Yet, PM_TRA_ estimates based on EURAD-CTM, which models mean concentrations within 1 km^2^, represent urban background concentrations in this area, rather than localized exposure contrasts that can be found, for example, near roads with high traffic (Zhu et al. 2002). Therefore, the IQR is an unsuitable exposure contrast for comparing estimated effects of source-specific PM in our study.

The estimated contributions of the local traffic and industry to total PM in the study area seem unexpectedly small (< 5% and < 11%, respectively). These numbers, however, are plausible, considering that secondary or transported particles from outside the Ruhr Area were disregarded. Long-range transport and formation of secondary particles in the atmosphere can contribute considerably, sometimes > 50% depending on the meteorological situation, to the particle mass concentration in North Rhine-Westphalia and the Ruhr Area (Hebbinghaus et al. 2009).

We observed some heterogeneity with regard to the estimated exposure effects, when adjusting for city. Although estimated effects of PM_TRA_ were robust toward the adjustment for city, estimated effects of PM_IND_ and PM_ALL_ increased considerably upon city adjustment. This finding might be a result of different spatial contrasts of specific particle concentrations within our study area, and certainly indicates the presence of residual confounding dependent on different exposure sources.

Analysis of effect modification suggested that the association between hs-CRP and PM_TRA_ was stronger in participants who were highly exposed to PM_IND_. This is consistent with the stronger associations estimated for participants living in Mülheim, where the concentrations of PM_IND_ (because of heavy industry in neighboring Duisburg) and PM_ALL_ were higher than in other regions of the HNR study area.

PM_TRA_ was not only more strongly associated with hs-CRP than PM_ALL_ or PM_IND_ when classified based on long-term exposure, but also when classified according to short- and medium-term time periods. We estimated stronger associations as PM_TRA_ exposure times increased. This could be due to more precise exposure estimation, or it could reflect a cumulative effect of PM_TRA_ on subclinical inflammation.

*Limitations and strengths*. One limitation of our study is that the approach of assessing source-specific air pollution is based on simulation runs and not on actual measurements. A second limitation is that we focused on fresh local emissions in this analysis, not taking transported emissions into account, which can contribute to the local concentration by > 50%, depending on the meteorological situation. One strength of our study is the large and well-characterized population-based, prospective cohort with repeated measures of hs-CRP and allowing comprehensive adjustment for confounding. Furthermore, we were able to take a first step into analyzing source-specific emissions with a novel method that can be applied to other sources as well. We were able to model traffic-specific and industry-specific PM independent of each other and therefore could estimate associations separately for these two major anthropogenic sources of particles. In addition, source-specific exposures were modeled with a fine temporal resolution (daily concentrations), allowing the construction of different short-, medium-, and long-term exposure indicators, depending on the research question.

## Conclusions

In summary, we estimated source-specific PM (PM_2.5_, PM_10_) exposures in a large population-based cohort using a novel approach based on the EURAD-CTM. Our results suggest that a 1-μg/m^3^ increase in long-term average exposure to fresh local traffic-specific PM was more strongly associated with hs-CRP, a marker of systemic inflammation, than a 1-μg/m^3^ increase in long-term total PM, independent of short-term average exposures. Associations with local industry-specific PM were slightly stronger than associations with total PM, but we were not able to detect significant differences. Future investigations will include the contribution of long-distance–transported source-specific emissions and different particle sizes.

## Supplemental Material

(119 KB) PDF
