# Supplementary material for: Association between Source-Specific Particulate Matter Air Pollution and hs-CRP: Local Traffic and Industrial Emissions
Source: Environ Health Perspect. 2014 Apr 22;122(7):703–10. doi: 10.1289/ehp.1307081 (PMC4080540; doi:10.1289/ehp.1307081)
Supplement: (119 KB) PDF [file ehp.1307081.s001.pdf]

Environmental Health Perspectives doi:10.1289/ehp.1307081

Supplemental Material

Association between Source-Specific Particulate Matter Air Pollution and hs-CRP: Local Traffic and Industrial Emissions

Frauke Hennig, Kateryna Fuks, Susanne Moebus, Gudrun Weinmayr, Michael Memmesheimer, Hermann Jakobs, Martina Bröcker-Preuss, Dagmar Führer-Sakel, Stefan Möhlenkamp, Raimund Erbel, Karl-Heinz Jöckel, and Barbara Hoffmann on behalf of the Heinz Nixdorf Recall Study Investigative Group

Air Pollution Exposure Assessment

We used the validated time dependent three-dimensional chemistry transport model European Air Pollution Dispersion model (EURAD-CTM) (Ebel et al. 1997; Hass et al. 1993; Memmesheimer et al. 2004; Schell et al. 2001) to predict daily mass concentrations of PM with an aerodynamic diameter ≤ 10 µm (PM_{10}) and ≤ 2.5 µm ($PM_{2.5}$) on a horizontal grid resolution of 1 km, which is the finest horizontal resolution for anthropogenic emission data in North-Rhine Westphalia provided by the North Rhine Westphalia State Agency for Nature, Environment and Consumer Protection (LANUV-NRW). The EURAD-CTM model is based on the fundamental physical principles of conservation of mass, momentum and energy. It combines the equations for fluid dynamics (e.g. used in models for numerical weather prediction) with the knowledge of chemical and physical transformations of atmospheric constituents in the earth's atmosphere to simulate transport, chemical transformation and deposition of air pollutants within a multi-layer, multi-species Eulerian grid system. The lowest layer is about 40 m high, while the upper boundary of the model is at an altitude of about 16 km. The multi-species Eulerian grid system used within the EURAD-CTM is based on sequential nesting of four horizontal grid sizes from Europe (grid size of 125 km) over central Europe (25 km), North Rhine-Westphalia in Germany (5 km) to (the south-western part of) the Ruhr area (Duisburg-Mülheim-Essen-Bochum) (1 km) (Büns et al. 2012, Memmesheimer et al. 2004). The method of sequential nesting is applied to include long range transport from the European scale towards North-Rhine-Westphalia and the Ruhr area (Memmesheimer et al, 2004). The vertical resolution is the same for all model nests.

Advanced numerical techniques are used to solve the system of partial differential equations with respect to appropriate input data as land use, topography and emissions of atmospheric

constituents due to anthropogenic activities and biogenic origin (e.g. in forests). The emission input is structured with respect to different source categories according to the Selected

Nomenclature for Sources of Air Pollution (SNAP nomenclature, SNAP-97), which e.g. includes traffic and industrial sources as different source categories. All emission input has been specified with a temporal resolution of one hour at each grid point in each nest of the model. Daily, weekly and seasonal variations in the emission input were considered. Due to the method of sequential nesting the long range transport and formation of atmospheric gases and particulate matter is included in the model, i.e. the formation of secondary particles in the atmosphere from primary emitted gaseous pollutants from NO2, SO2, NH3 and volatile organic compounds during the transport (Schell et al., 2001). Long range transport and formation of secondary particles in the atmosphere can contribute considerably, partially more than 50% depending on the meteorological situation, to the particle mass concentration in North-Rhine-Westphalia and the Ruhr area (Hebbinghaus et al., 2009).

Output of the EURAD-CTM calculations include the meteorological variables temperature, radiation, precipitation, wind and turbulence parameters as well as a set of chemical compounds such as atmospheric particle mass, number density and particle size distribution and concentration of atmospheric gases, photo oxidants and a set of volatile organic compounds on a daily basis.

To evaluate the additional contribution of regionally transported traffic emissions, we implemented a model excluding traffic sources of all four sequential nests (125 km-25 km-5 km-1 km) for PM_{10} and compared resulting mean PM_{10} concentrations with the mean PM_{10} concentrations from only local suppression within the Ruhr Area (1 km) on arbitrary days (in

2001) in winter, spring, summer and fall and for the whole year 2001. This comparison between PM_{10} concentrations in the Ruhr Area after suppression of local road traffic emissions within the Ruhr Area and after total suppression of road traffic emissions in Europe, central-Europe, North-Rhine-Westphalia and Ruhr Area showed that PM_{10} concentrations differed only slightly. Differences in concentrations were slightly larger during winter and summer and in the south and west of Ruhr Area. Comparing the mean value of yearly concentrations, differences were slightly larger in the western part of Ruhr Area (data not shown). This analysis showed that traffic-specific PM concentrations are slightly influenced by long-distance transport of particles. We expect that local industry-specific PM concentrations are also influenced by long-distance transport of particles as well or maybe even more.

References

Büns C, Klemm O, Wurzler S, Hebbinghaus H, Steckelbach I, Friesel J, et al. 2012. Comparison of four years of air pollution data with a mesoscale model. Atmos. Res. 118:404–417; doi:10.1016/j.atmosres.2012.07.009.

Ebel A, Elbern H, Feldmann H, Jakobs HJ, Kessler C, Memmesheimer M et al. 1997. Air pollution studies with the EURD model system (3). Report 120. Institute of Geophysics and Meteorology, University of Cologne.

Hass H, Ebel A, Feldmann H, Jakobs HJ, Memmesheimer M,1993. Evaluation studies with a regional chemical transport model (EURAD) using air quality data from the EMEP monitoring network. Atmos. Environ. 27A, 867–887.

Hebbinghaus H, Wurzler S, Friese E, Jakobs HJ, Kessler C, Ebel A. 2009. Determination of the contribution of different groups of emission sources on the concentration of PM10 , PM2.5 , and NO2 in North Rhine-Westphalia – a whodunnit. 28293. European Aerosol Conference 2009, Karlsruhe, Abstract T031A03, 2009.

Memmesheimer M, Friese E, Ebel A, Jakobs HJ, Feldmann H, Kessler C et al. 2004. Long-term simulations of particulate matter in Europe on different scales using sequential nesting of a regional model. Int. J. Environ. Pollut. 22, 108–132.

Schell B, Ackermann IJ, Hass H, Binkowski FS, Ebel A, 2001. Modeling the formation of secondary organic aerosol within a comprehensive air quality modeling system. J. Geophys. Res. 106, 28275–28293.

Selected Nomenclature for Air Pollution (SNAP-97).
